# The distribution of bacterial doubling times in the wild

**DOI:** 10.1098/rspb.2018.0789

**Published:** 2018-06-13

**Authors:** Beth Gibson, Daniel J. Wilson, Edward Feil, Adam Eyre-Walker

**Affiliations:** 1 School of Life Sciences, University of Sussex, Brighton BN1 9QG, UK; 2 Nuffield Department of Medicine, University of Oxford, John Radcliffe Hospital, Oxford OX3 9DU, UK; 3 The Milner Centre for Evolution, Department of Biology and Biochemistry, University of Bath, Claverton Down, Bath, BA2 7AY, UK

**Keywords:** generation time, mutation rates, bacteria

## Abstract

Generation time varies widely across organisms and is an important factor in the life cycle, life history and evolution of organisms. Although the doubling time (DT) has been estimated for many bacteria in the laboratory, it is nearly impossible to directly measure it in the natural environment. However, an estimate can be obtained by measuring the rate at which bacteria accumulate mutations per year in the wild and the rate at which they mutate per generation in the laboratory. If we assume the mutation rate per generation is the same in the wild and in the laboratory, and that all mutations in the wild are neutral, an assumption that we show is not very important, then an estimate of the DT can be obtained by dividing the latter by the former. We estimate the DT for five species of bacteria for which we have both an accumulation and a mutation rate estimate. We also infer the distribution of DTs across all bacteria from the distribution of the accumulation and mutation rates. Both analyses suggest that DTs for bacteria in the wild are substantially greater than those in the laboratory, that they vary by orders of magnitude between different species of bacteria and that a substantial fraction of bacteria double very slowly in the wild.

## 1. Introduction

The bacterium *Escherichia coli* can divide every 20 min in the laboratory under aerobic, nutrient-rich conditions. But how often does it divide in its natural environment in the gut, under anaerobic conditions where it probably spends much of its time close to starvation? And what do we make of a bacterium, such as *Syntrophobacter fumaroxidans*, which only doubles in the laboratory every 140 h [1]. Does this reflect a slow doubling time (DT) in the wild, or our inability to provide the conditions under which it can replicate rapidly?

Estimating the generation time is difficult for most bacteria in their natural environment and very few estimates are available. The DT for intestinal bacteria has been estimated in several mammals by assaying the quantity of bacteria in the gut and faeces. Assuming no cell death Gibbons & Kapsimalis [2] estimate the DT for all bacteria in the gut to be 48, 17 and 5.8 h in hamster, guinea pig and mouse, respectively. More recently Yang *et al*. [3] have shown that the DT of *Pseudomonas aeruginosa* is correlated to cellular ribosomal content *in vitro* and have used this to estimate the DT *in vivo* in a cystic fibrosis (CF) patient to be between 1.9 and 2.4 h.

Although there are very few estimates of the generation time in bacteria, this quantity is important for understanding bacterial population dynamics. Here we use an indirect method to estimate the DT that uses two sources of information. First, we can measure the rate at which a bacterial species accumulates mutations in its natural environment through time using temporarily sampled data [4], or concurrent samples from a population with a known date of origin. We refer to this quantity as the accumulation rate, to differentiate it from the mutation rate, and the substitution rate, the rate at which mutations spread through a species to fixation. If we assume that all mutations in the wild are neutral, an assumption that we show to be relatively unimportant, then the accumulation rate is an estimate of the mutation rate per year, *u_y_*. Second, we can estimate the rate of mutation per generation, *u_g_*, in the laboratory using a mutation accumulation experiment and whole-genome sequencing, or through fluctuation tests. If we assume that the mutation rate per generation is the same in the wild and in the laboratory, an assumption we discuss further below, then if we divide the accumulation rate per year in the wild by the mutation rate per generation in the laboratory, we can estimate the number of generations that the bacterium goes through in the wild and hence the DT (DT = 8760 × *u_g_*/*u_y_*, where 8760 is the number of hours per year).

## 2. Results

The accumulation rate in the wild and the mutation rate in the laboratory have been estimated for 34 and 26 bacterial species, respectively (electronic supplementary material, tables S1, S2); we only consider mutation rate estimates from mutation accumulation experiments, because estimates from fluctuation tests are subject to substantial sampling error and unknown bias, and we exclude estimates from hypermutable strains. For five species, *E. coli, P. aeruginosa, Salmonella enterica, Staphylococcus aureus* and *Vibrio cholerae*, we have both an accumulation and a mutation rate estimate and hence can estimate the DT. Among these five species we find our DT estimates vary from 1.1 h in *V. cholerae* to 25 h in *S. enterica* (table 1). In all cases the estimated DT in the wild is greater than that of the bacterium in the laboratory. For example, *E. coli* can double every 20 min in the laboratory but we estimate that it only doubles every 15 h in the wild.

**Table 1.** Doubling time estimates (hours) for those species for which we have both an estimate of the accumulation and mutation rate. Accumulation rate (AR) references—(1) [5]; (2) [6,7]; (3) [8–12]; (4) [13–23]; (5) [8,24]. Mutation rate (MR) references—(6) [25]; (7) [26]; (8) [27]; (9) [28]; (10) [29].

species	accumulation rate per site per year	mutation rate per site per generation	DT (h) (s.e.)	laboratory DT (h)	ratio	AR ref.	MR ref.
*Escherichia coli*	1.44 × 10^−7^	2.54 × 10^−10^	15 (7.7)	0.33	45	1	6
*Pseudomonas aeruginosa*	3.03 × 10^−7^	7.92 × 10^−11^	2.3 (0.77)	0.5	4.6	2	7
*Salmonella enterica*	2.50 × 10^−7^	7.00 × 10^−10^	25 (7.9)	0.5	50	3	8
*Staphylococcus aureus*	2.05 × 10^−6^	4.38 × 10^−10^	1.87 (0.98)	0.4	4.7	4	9
*Vibrio cholerae*	8.30 × 10^−7^	1.07 × 10^−10^	1.1 (0.26)	0.66	1.7	5	10

**Figure 1.**
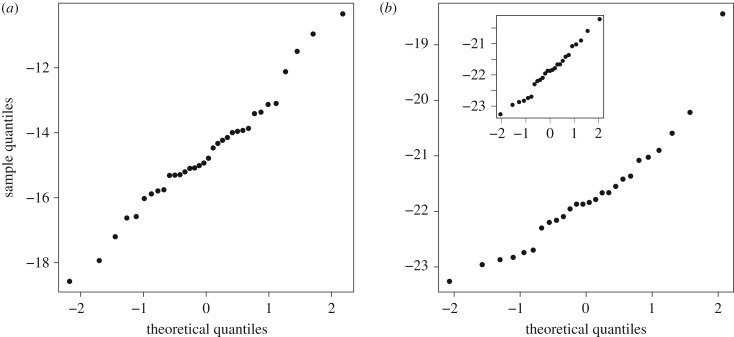
Normal *Q*–*Q* plots for the log of (*a*) accumulation and (*b*) mutation rate data. The main plot in (*b*) includes all 26 mutation rate estimates and the insert excludes *Mesoplasma florum* estimate.

In theory, it might be possible to estimate the DT in those bacteria for which we have either an accumulation or mutation rate estimate, but not both, by finding factors that correlate with either rate and using those factors to predict the rates. Unfortunately, we have been unable to find any factor that correlates sufficiently well to be usefully predictive. It has been suggested that the mutation rate is correlated to genome size in microbes [30] but, the current evidence for this correlation is very weak, and depends upon the estimate from *Mesoplasma florum* (*r* = −0.68, *p* < 0.001 with *M. florum* and *r* = −0.39, *p* = 0.053 without *M. florum*) (electronic supplementary material, figure S1) [31]. However, we can use the accumulation and mutation rate estimates to estimate the distribution of DTs across bacteria if we assume that there is no phylogenetic non-independence in the mutation and accumulation data, an assumption we address below. We can estimate the distribution of DTs by fitting distributions to the accumulation and mutation rate data, using maximum likelihood, and then dividing one distribution by the other. We assume that both variables are lognormally distributed, an assumption that is supported by *Q*–*Q* plots with the exception of the mutation rate per generation in *M. florum*, which is a clear outlier (figure 1). We repeated all our analyses with and without *M. florum*.

If the accumulation and mutation rate data are lognormally distributed then the distribution of DT is also lognormally distributed with a mean of log_e_(8760) + *m_g_* − *m_y_* and a variance of *v_g_* + *v_y_* − 2Cov(*g,y*), where 8760 is the number of hours per year and *m_g_*, *m_y_*, *v_g_* and *v_y_* are the mean and variance of the lognormal distributions fitted to the mutation (subscript *g)* and accumulation (subscript *y*) rates. *Cov*(*g,y*) is the covariance between the accumulation and mutation rates. We might expect that species with higher mutation rates also have higher accumulation rates, because the accumulation rate is expected to depend on the mutation rate, but the correlation between the two will depend upon how variable the DT and other factors, such as the strength of selection, are between bacteria. The observed correlation between the log accumulation rate and log mutation rate is 0.077, but there are only five data points, so the 95% confidence intervals on this estimate encompass almost all possible values (−0.86 to 0.89). We explore different levels of the correlation between the accumulation and mutation rates; it should be noted that *Cov*(*g,y*) can be expressed as Sqrt(*v_g_ v_y_*) *Corr*(*g,y*) where *Corr*(*g,y*) is the correlation between the two variables.

**Figure 2.**
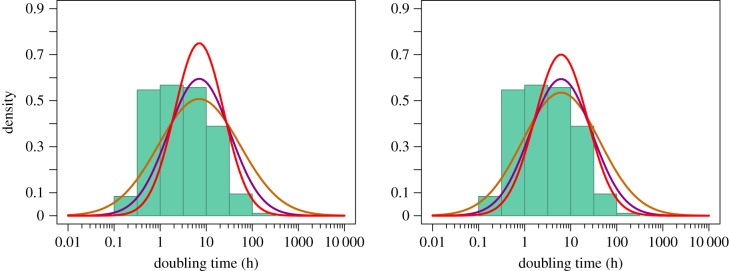
The distribution of DTs among bacteria inferred assuming different levels of correlation between the accumulation and mutation rates—orange *r* = 0, purple *r* = 0.5 and red *r* = 0.75. We also show the distribution of laboratory DTs (green histogram) from a compilation of over 200 species made by Vieira-Silva & Rocha [32]. In (*a*) we include all mutation rate estimates and in (*b*) we exclude the mutation rate estimate for *Mesoplasma florum*. (Online version in colour.)

The distribution of DTs in the wild inferred using our method is shown in figure 2. We infer the median DT to be 7.04 h, but there is considerable spread around this even when the accumulation and mutation rates are strongly correlated (figure 2*a*); as the correlation increases so the variance in DTs decreases, but the median remains unaffected. The analysis suggests that most bacteria have DTs of between 1 and 100 h but there are substantial numbers with DTs beyond these limits. For example, even if we assume that the correlation between the accumulation and mutation rate is 0.5 we infer that 10% of bacteria have a DT of faster than 1 h in the wild and 4.2% have a DT slower than 100 h in the wild. If we remove the *M. florum* mutation rate estimate from the analysis the median doubling is slightly lower at 6.16 h, but there is almost as much variation as when this bacterium is included; at a correlation is 0.5 we infer that 12% of bacteria have a DT faster than 1 h in the wild and 3.5% have a DT slower than 100 h.

**Figure 3.**
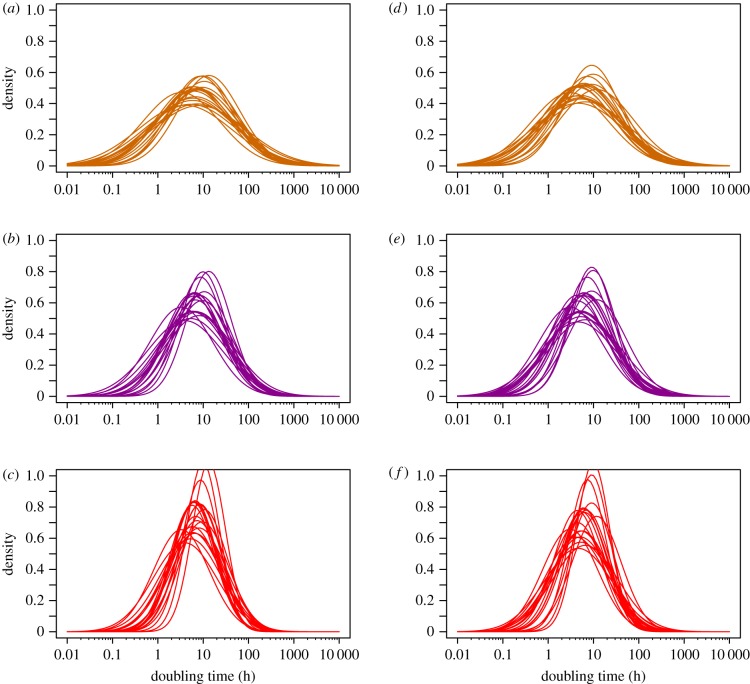
DT distributions inferred by bootstrapping the accumulation and mutation rate data and refitting the lognormal distributions to both datasets. Each plot shows 20 bootstrap DT distributions assuming different levels of correlation between the accumulation and mutation rates—orange *r* = 0, purple *r* = 0.5 and red *r* = 0.75. (*a*–*c*) Include all mutation rate estimates and (*d*–*f*) show the analysis after removal of the *Mesoplasma florum* mutation rate estimate. (Online version in colour.)

To investigate how robust these conclusions are to statistical sampling, we bootstrapped the accumulation and mutation rate estimates, refit the lognormal distributions and reinferred the distribution of DT. The 95% confidence intervals for the median are quite broad at 3.4 to 14.2 h (3.1 to 11.3 h if we exclude *M. florum*). However, all bootstrapped distributions show substantial variation in the DT with a substantial fraction of bacteria with long DTs and also some with very short DTs (figure 3).

**Figure 4.**
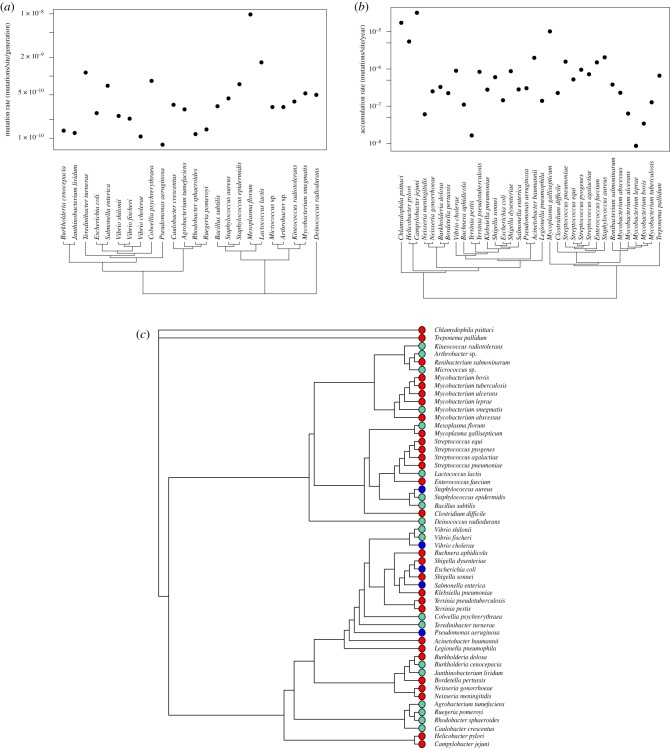
(*a*) 16S rRNA phylogeny and mutation rate estimates for 24 species of bacteria (two species are excluded because of erroneous positioning on the phylogeny—see electronic supplementary material, figure S2A,B for details). (*b*) 16S rRNA phylogeny and accumulation rate estimates for 34 species of bacteria. (*c*) 16S rRNA phylogeny combining species for which we have an estimate of the mutation rate and/or accumulation rate. Coloured dots indicate which kind of information each species provides—red, accumulation rate; green,mutation rate and blue, both a mutation rate and an accumulation rate. (Online version in colour.)

We have assumed that there is no phylogenetic inertia within the accumulation and mutation rate estimates. To test whether this is the case we constructed a phylogenetic tree using 16S rRNA sequences and applied the tests of Pagel [33] and Blomberg *et al.* [34]. Both the accumulation and mutation rate data show some evidence of phylogenetic signal. For the accumulation data, Pagel's *λ* = 0.68 (*p* = 0.001) and Blomberg *et al*.'s *K* = 0.0005 (*p* = 0.35); and for the mutation rate data Pagel's *λ* = 0.88 (*p* = 0.026) and Blomberg *et al*.'s *K* = 0.5 (*p* = 0.009). We also find some evidence that the data depart from a Brownian motion model using Pagel's test (i.e. *λ* is significantly less than one) for the accumulation data (*p* < 0.001) but not the mutation rate data (*p* = 0.094); i.e. the accumulation rates are more different than we would expect from their phylogeny and a Brownian motion model. A visual inspection of the data suggests that the phylogenetic signal is largely contributed by species that are closely related, rather than deeper phylogenetic levels (figure 4*a*,*b*) and species for which we have accumulation and mutation rate estimates are interspersed with one another on the phylogenetic tree (figure 4*c*). It, therefore, seems unlikely that phylogenetic inertia will influence our results.

It is of interest to compare the distribution of DTs in the wild to the distribution of laboratory DTs (figure 1). The distributions are different in two respects. First, the median laboratory DT of 3 h is less than half the median wild DT of 7.04 h (6.16 h without *M. florum*); the two are significantly different (*p* = 0.012 with *M. florum* and *p* = 0.016 without *M. florum*, inferred by bootstrapping each dataset and recalculating the medians). Second, many more bacteria are inferred to have long DTs in the wild than in the laboratory.

## 3. Discussion

The DT of most bacteria in their natural environment is not known. We have used estimates of the rate at which bacteria accumulate mutations in their natural environment and estimates of the rate at which they mutate in the laboratory to estimate the DT for several bacteria and infer the distribution of DTs across bacteria. We estimate that DT are generally longer in the wild than in the laboratory, but critically we also infer that DTs vary by several orders of magnitude between bacterial species and that many bacteria have very slow DTs in their natural environment.

The method, by which we have inferred the DT in the wild, makes three important assumptions. We assume that the mutation rate per generation is the same in the laboratory and in the wild. However, it seems likely that bacteria in the wild will have a higher mutation rate per generation than those in the laboratory for two reasons. First, bacteria in the wild are likely to be stressed and this can be expected to elevate the mutation rate [35–39]. Second, if we assume that DTs are longer in the wild than the laboratory then we expect the mutation rate per generation to be higher in the wild than in the laboratory because some mutational processes do not depend upon DNA replication. The relative contribution of replication dependent and independent mutational mechanisms to the overall mutation rate is unknown. Rates of substitution are higher in Firmicutes that do not undergo sporulation suggesting that replication is a source of mutations in this group of bacteria [40]. However, rates of mutation accumulation seem to be similar in latent versus active infections of *Mycobacterium tuberculosis*, suggesting that replication independent mutations might dominate in this bacterium [41,42].

The second major assumption is that the rate at which mutations accumulate in the wild is equal to the mutation rate per year; in effect, we are assuming that all mutations are effectively neutral, at least over the time frame in which they are assayed (or that some are inviable, but the same proportion are inviable in the wild and the laboratory). In those accumulation rate studies, in which they have been studied separately, non-synonymous mutations accumulate more slowly than synonymous mutations; relative rates vary from 0.13 to 0.8, with a mean of 0.57 (electronic supplementary material, table S3). There is no correlation between the time frame over which the estimate was made and the ratio of non-synonymous and synonymous accumulation rates (*r* = 0.2, *p* = 0.53). We did not attempt to control for selection because the relative rates of synonymous and non-synonymous accumulation are only available for a few species, and the relative rates vary between species. However, we can estimate the degree to which more selection against deleterious non-synonymous accumulations in the wild causes the DT to be underestimated as follows. The observed rate at which mutations accumulate in a bacterial lineage is3.1
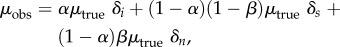
where *α* is the proportion of the genome that is non-coding and *β* is the proportion of mutations in protein coding sequence that are non-synonymous. *δ_x_* is the proportion of mutations of class *x* (*i* is intergenic, *s* is synonymous and *n* is non-synonymous) that are effectively neutral. *α* and *β* are approximately 0.15 and 0.7, respectively, in our dataset. Although there is selection on synonymous codon use in many bacteria [43], selection appears to be weak [44] we, therefore, assume that *δ_s_* = 1. This implies, from the rate at which non-synonymous mutations accumulate relative to synonymous mutations, that *δ_n_* = 0.6. A recent analysis of intergenic regions in several species of bacteria has concluded that selection is weaker in intergenic regions than at non-synonymous sites; we, therefore, assume that *δ_i_* = 0.8 [45]. Using these estimates suggests that selection leads us to underestimate the true mutation rate per year in the wild by approximately 27%; this in turn means we have over-estimated the DT by approximately 37%, a relatively small effect. To investigate how sensitive this estimate is to the parameters in equation 1, we varied each of them in turn (electronic supplementary material, table S4). We find that the observed mutation rate is most sensitive to selection on synonymous codon use, because if there is selection on synonymous codon use this also affects our estimates of selection at non-synonymous sites and in intergenic. For example, if selection on synonymous codon use depressed the synonymous accumulation rate by 0.5 this would lead to an underestimate of the mutation rate of 63%, which would in turn have led to a 2.7-fold over-estimate of the DT.

Finally, although each study attempted to remove single nucleotide polymorphisms (SNPs) that had arisen by recombination, it is possible that some are still present in the data. Recombinant SNPs can have two effects. First, if they have recombined from outside the clade, they inflate the accumulation rate estimate and hence lead to an underestimate of the DT. Second, if there is recombination within a clade, they affect the phylogeny and potentially lead to the root of the tree being estimated as younger than it should be. This will lead to an overestimate of the DT.

It is important to appreciate that our method estimates an average DT within a particular environment that the bacteria were sampled from. The bacterium may go through periods of quiescence interspersed with periods of growth.

Despite the assumptions we have made in our method, our estimate of the DT of *Pseudomonas aruginosa* of 2.3 h in a CF patient is very similar to that independently estimated using the ribosomal content of cells of between 1.9 and 2.4 h [3]. There is also independent evidence that there are some bacteria that divide slowly in their natural environment. The aphid symbiont *Buchnera aphidicola* is estimated to double every 175–292 h in its host [46,47], and *Mycobacterium leprae* doubles every 300–600 h on mouse footpads [48–50], not its natural environment, but one that is probably similar to the human skin. Furthermore, in a recent selection experiment, Avrani *et al*. [51] found that several *E. coli* populations, which were starved of resources, accumulated mutations in the core RNA polymerase gene. These mutations caused these strains to divide more slowly than unmutated strains when resources were plentiful. Interestingly these same mutations are found at high frequency in unculturable bacteria, suggesting that there is a class of slow growing bacteria in the environment that are adapted to starvation.

Korem *et al*. [52] have recently proposed a general method by which the DT can be potentially estimated. They note that actively replicating bacterial cells have two or more copies of the chromosome near the origin of replication but only one copy near the terminus, if cell division occurs rapidly after the completion of DNA replication. Using next-generation sequencing, they show that it is possible to assay this signal and that the ratio of sequencing depth near the origin and terminus is correlated to bacterial growth rates *in vivo*. Brown *et al*. [53] have extended the method to bacteria without a reference genome and/or those without a known origin and terminus of replication. In principle, these measures of cells performing DNA replication could be used to estimate the DT of bacteria in the wild. However, it is unclear how or whether the methods can be calibrated. Both Korem *et al*. (2015) and Brown *et al*. (2016) find that their replication measures have a median of approximately 1.3 across bacteria in the human gut. However, a value of 1.3 translates into different relative and absolute values of the DT in the two studies. Brown *et al*. [53] show that their measure of replication, iRep, is highly correlated to Korem *et al*.'s [52] measure, PTR, for data from *Lactobacillus gasseri*; the equation relating the two statistics is iRep = −0.75 + 2 PTR. Hence, when PTR = 1.3, iRep = 1.85 and when iRep = 1.3, PTR = 1.03. The two methods are not consistent. They also yield very different estimates for the absolute DT. Korem *et al*. [52] show that PTR is highly correlated to the growth rate of *E. coli* grown in a chemostat. If we assume that the relationship between PTR and growth rate is the same across bacteria *in vivo* and *in vitro*, then this implies that the median DT for the human microbiome is approximately 2.5 h. By contrast, Brown *et al*. [53] estimate the growth rate of *Klebsiella oxytoca* to be 19.7 h in a new-born baby using faecal counts and find that this population has an iRep value of approximately 1.77. This value is greater than the vast majority of bacteria in the human microbiome and bacteria in the Candidate Phyla Radiation, suggesting that most bacteria in these two communities replicate very slowly. These discrepancies between the two methods suggest that it may not be easy to calibrate the PTR and iRep methods to yield estimates of the DT across bacteria.

Finally, how should we interpret our results for the five focal species in the context of what is known of their ecology? *Vibrio cholerae* displays the shortest DT of 1.1 h. *Vibrio* species are ubiquitous in estuarine and marine environments [54]. They are known to have very short generation times in culture, the shortest being *Vibrio natriegens* of just 9.8 min [55]. In the wild they can exploit a wide range of carbon and energy sources, and as such have been termed ‘opportunitrophs’ [56]. Natural *Vibrio* communities do not grow at an accelerated rate continuously, but can exist for long periods in a semi-dormant state punctuated by rapid pulses of high growth rates [57], or blooms [58], when conditions are favourable. It has also been argued that the unusual division of *Vibrio* genomes into two chromosomes facilitates more rapid growth [59]. By pointing to a very short DT in *V. cholerae*, our analysis is, therefore, consistent with what is known of the ecology of this species.

*Staphylococcus aureus* is predominantly found on animals and humans and inhabits various body parts, including the skin and upper respiratory tract [60]. It can cause infection of the skin and soft tissue as well as bacteraemia [61]. *Staphylococcus aureus* exhibits a range of modes of growth, some of which may to allow it to survive stress and antimicrobials while in its host. For instance, small subpopulations can adopt a slow-growing, quasi-dormant lifestyle, either in a multicellular biofilm or as small colony variants (SCVs) or persister cells [62]. Our short DT of 1.8 h suggests this is not the typical state for *S. aureus* in the wild, which is not surprising considering the incidence of SCVs in clinical samples is fairly low, between 1 and 30% [63].

*Pseudomonas aeruginosa* can inhabit a wide variety of environments, including soil, water, plants and animals. Like our other focal species, it is an opportunistic pathogen and can also infect humans, especially those with compromised immune systems, such as patients with CF. In this context infection is chronic. Parallel evolution, the differential regulation of genes which allow it to evade the host immune system and resist antibiotic treatment during infection [64], and evidence of positive selection [65] suggests *P. aeruginosa* can adapt to the lungs of individuals with CF for its long-term survival. It is known to actively grow in sputum [3], where it uses the available nutrition which supports its growth to high population densities [66]. Its ability to adapt and actively grow in the CF sputum is consistent with its relatively short DT of 2.3 h, especially considering this is the environment in which the accumulation rate was measured and matches that estimated by Yang *et al.* [3].

*Escherichia coli* and *S. enterica* primarily reside in the lower intestine of humans and animals, but can also survive in the environment. Although *E. coli* is commonly recovered from environmental samples, it is not thought able to grow or survive for prolonged periods outside of the guts of warm blooded animals, except in tropical regions where conditions are more favourable [67], although some phylogenetically distinct strains appear to reproduce and survive well in the environment [68]. In contrast, *Salmonella* is also an enteric colonizer of cold-blooded animals, in particular reptiles, is better adapted than *E. coli* at surviving and growing in environmental niches. For example, *Salmonella* can survive and grow for at least a year in soil [69], whereas *E. coli* can survive for only a few days [70]. Although these secondary niches may play a greater role in *Salmonella* than in *E. coli*, it remains the case the growth rates in the environment will be much lower than those in a gut. Therefore, the increased tenacity of *Salmonella* in non-host environments compared to *E. coli* might help to explain the slower DT in this species.

In summary, the availability of accumulation and mutation rate estimates allows us to infer the DT for bacteria in the wild, and the distribution of wild DTs across bacterial species. These DT estimates are likely to be underestimates because the mutation rate per generation is expected to be higher in the wild than in the laboratory, and some mutations are not generated by DNA replication. Our analysis, therefore, suggests that DTs in the wild are typically longer than those in the laboratory, that they vary considerably between bacterial species and that a substantial proportion of species have very long DTs in the wild.

## 4. Material and methods

We compiled estimates of the accumulation and mutation rate of bacteria from the literature. We only used mutation rate estimates that came from a mutation accumulation experiment with whole-genome sequencing. If we had multiple estimates of the mutation rate we summed the number of mutations across the mutation accumulation experiments and divided this by the product of the genome size and the number of generations that were assayed. We averaged the accumulation rate estimates where we had multiple estimates from the same species. We recalculated the accumulation rates in two cases in which the number of accumulated mutations had been divided by an incorrect number of years: *E. coli* [5] and *Helicobacter pylori* [71]. For *E. coli*, we reestimated the accumulation rate using BEAST by constructing sequences of the SNPs reported in the paper and the isolation dates. For, *Helicobacter pylori* the 3-year and 16-year strains appear to form a clade to the exclusion of the 0-year strain because they share some differences from the 0-year strain. If the number of substitutions that have accumulated between the common ancestor of the 3-year and 16-year strain and each of the two strains are *S*_3_ and *S*_16_, respectively, then the rate of accumulation can be estimated as (*S*_16_ *− S*_3_)/(13 years × genome size). For the isolates NQ1707 and NQ4060 we have estimated the accumulation rate to be 5 × 10^−6^ and for NQ1671 and NQ4191 5.9 × 10^−6^. We excluded some accumulation rate estimates for a variety of reasons. We only considered accumulation rates sampled over an historical time frame of at most 1500 years. Most of our estimates of the accumulation rate are for all sites in the genome, so we excluded cases in which only the synonymous accumulation rate was given. We also excluded accumulation rates from hypermutable strains. Accumulation and mutation rate estimates used in the analysis are given in electronic supplementary material, tables S1 and S2, respectively.

The estimate of the standard error associated with our estimate of the DT was obtained using the standard formula for the variance of a ratio: *V*(*x/y*) = (*M*(*x*)/*M*(*y*))^2^(*V*(*x*)/*M*(*x*)^2^ + *V*(*y*)/*M*(*y*)^2^) where *M* and *V* are the mean and variance of *x* and *y*. The variance for the accumulation rate was either the variance between multiple estimates of the accumulation rate if they were available, or the variance associated with the estimate if there was only a single estimate. The variance associated with the mutation rate was derived by assuming that the number of mutations was Poisson distributed.

We fit lognormal distributions to the accumulation and mutation rate data by taking the log_e_ of the values and then fitting a normal distribution by maximum likelihood using the *FindDistributionParameters* in *Mathematica*. Normal *Q*–*Q* plots for the accumulation and mutation rate data were produced using the qqnorm function in R version 1.0.143. In fitting these distributions, we have not taken into account the sampling error associated with the accumulation and mutation rate estimates. However, these sampling errors are small compared to the variance between species: for the accumulation rates the variance between species is 3.9 × 10^−11^ and the average error variance is an order of magnitude smaller at 3.6 × 10^−12^; for the mutation rate data, the variance between species is 7.5 × 10^−18^ and the average variance associated with sampling is more than two orders of magnitude smaller at 1.8 × 10^−20^. Note that we cannot perform these comparisons of variances on a log-scale because we do not have variance estimates for the log accumulation and mutation rates.

To estimate phylogenetic signal in the accumulation and mutation rates we generated phylogenetic trees for each set of species in the two datasets. 16S rRNA sequences were downloaded from the NCBI genome database (https://www.ncbi.nlm.nih.gov/genome/) and aligned using MUSCLE [72] performed in Geneious version 10.0.9 (http://www.geneious.com, Kearse *et al*. [73]). From these alignments, maximum-likelihood trees were constructed in RAxML [74] and integrated into the tests of Pagel [33] and Blomberg *et al*. [34] to the log_10_ (accumulation rates) and log_10_ (mutation rates) implemented in the phylosig function in the R package phytools v.0.6 [75]. For the mutation rate dataset two species were excluded because of erroneous positioning on the phylogeny. See electronic supplementary material, figure S2A,B for details.

## Supplementary Material

Supplementary Tables and Figures

## Supplementary Material

Electronic supplementary material (ESM) titles and captions

## Data accessibility

All relevant data for this work are available as electronic supplementary material accompanying this article.
