# Supplementary material for: The distribution of bacterial doubling times in the wild
Source: Proc Biol Sci. 2018 Jun 13;285(1880):20180789. doi: 10.1098/rspb.2018.0789 (PMC6015860; doi:10.1098/rspb.2018.0789)
Supplement: Supplementary Tables and Figures [file rspb20180789supp1.pdf]

Supplementary Figures and Tables

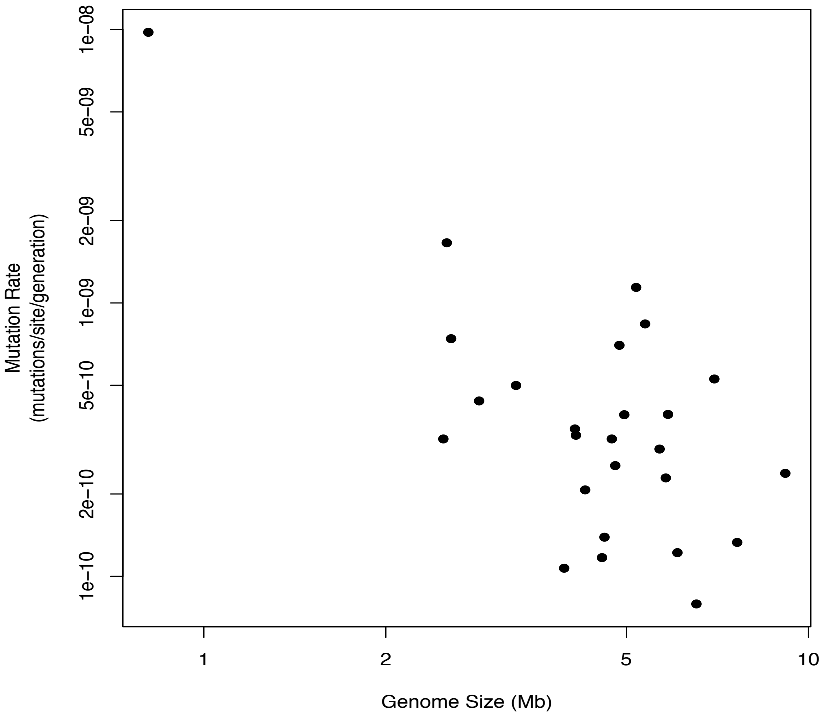

Supplementary Figure 1. Mutation rate vs genome size plotted for 26 species of bacteria.

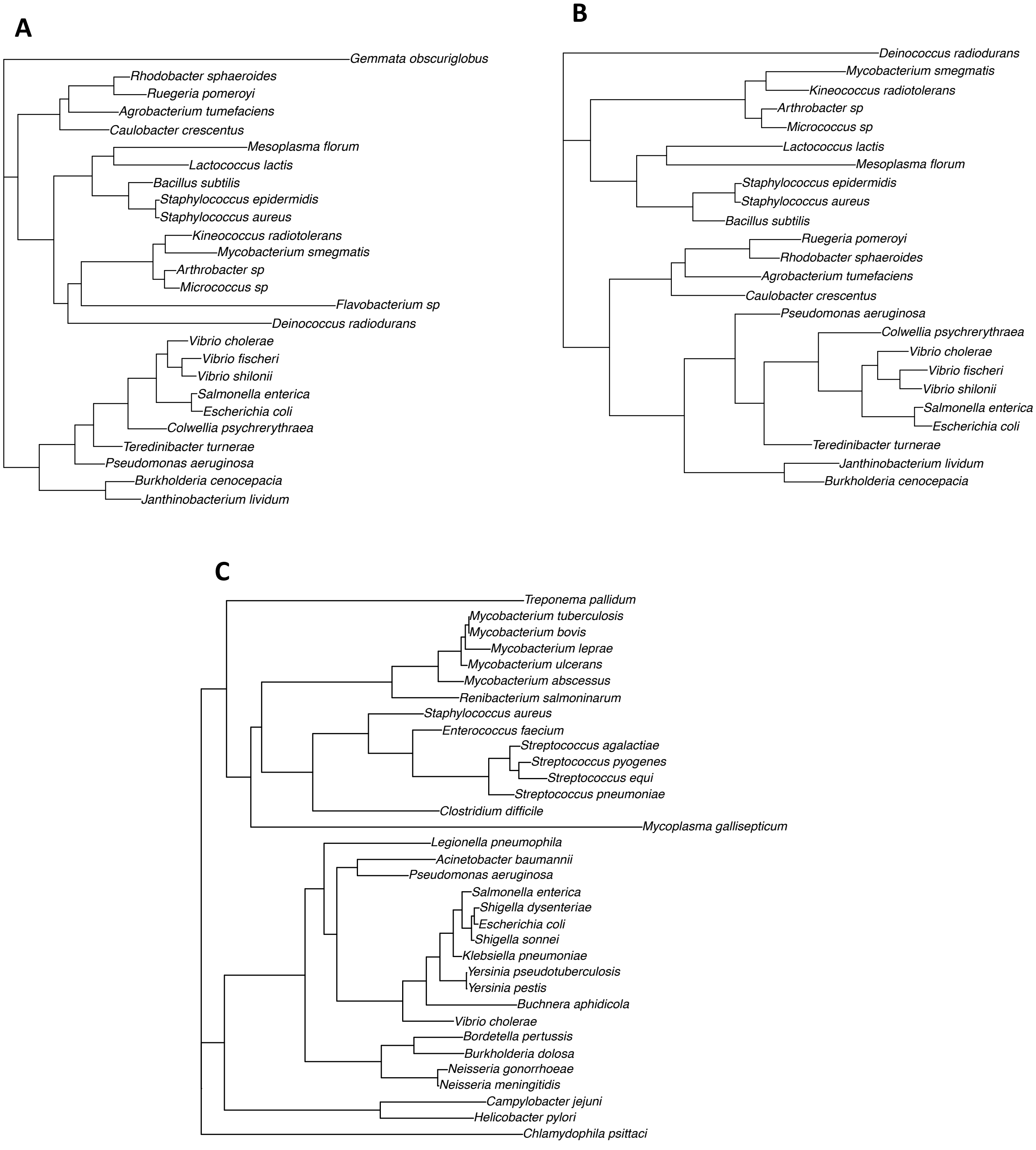

Supplementary Figure 2. 16s rRNA phylogenies for species for which we have a mutation rate estimate (A,B) and an accumulation rate (C). When all 26 species are included for the mutation rate data (A) Flavobacterium sp and the Alphaproteobacteria are erroneously positioned with the gram positive bacteria. This is resolved after exclusion of Flavobacterium sp and Gemmata obscuriglobus. (B).

Species	subgroup	Accumulation Rate (x10^{-7})	Included/ excluded/ recalculated	Reason for exclusion/recalculation	Reference
Acinetobacter baumannii	GC1	15.00	Included		[1]
Acinetobacter baumannii	GC2	24.70	Included		[2]
Bordetella pertussis		2.24	Included		[3]
Buchnera aphidicola		1.10	Included		[4]
Buchnera aphidicola		0.09	Excluded	Very long divergence (50 million years)	[5]
Burkholderia dolosa		3.28	Included		[6]
Campylobacter jejuni		323.00	Included		[7]
Chlamydia psittaci		174.00	Included		[8]
Clostridium difficile		3.20	Included		[9]
Clostridium difficile	27	1.70	Included		[10]
Clostridium difficile	027/BI/NAP1	1.88	Included		[11]
Enterococcus faecium	ST17/ST252	15.00	Included		[12]
Escherichia coli		1.44	Recalculated	Unsure about the rationale related to timepoints used in the calculation. We recalculated by running SNP alignment through BEAST	[13]
Helicobacter pylori		410.00	Excluded	SNPs might be recombinant	[14]
Helicobacter pylori		29.35	Excluded	Upper limit on estimate of the divergence time is arbitrary	[15]
Helicobacter pylori		138.00	Excluded	Synonymous rate	[16]
Helicobacter pylori		54.5	Recalculated	Cannot be sure that 3yr isolates are a direct descendant of 0yr isolates	[17]
Klebsiella pneumoniae	CC258 Clade1	2.56	Included		[18]
Klebsiella pneumoniae	CC258 Clade2	2.99	Included		[18]
Legionella pneumophilia		1.39	Included		[19]
Mycobacterium abscessus	subsp abscessus	3.63	Included		[20]
Mycobacterium abscessus	subsp massiliense	0.95	Included		[20]

Mycobacterium bovis		0.34	Included		[21]
Mycobacterium leprae		0.09	Included		[22]
Mycobacterium tuberculosis		0.49	Included		[23]
Mycobacterium tuberculosis		1.80	Included		[24]
Mycobacterium tuberculosis		1.14	Included		[25]
Mycobacterium tuberculosis		1.93	Included		[18]
Mycobacterium tuberculosis		1.00	Included		[26]
Mycobacterium ulcerans		0.63	Included		[27]
Mycoplasma gallisepticum		102.00	Included		[28]
Neisseria gonorrhoeae		2.50	Included		[29]
Neisseria meningitidis		0.61	Included		[18]
Pseudomonas aeruginosa	DK2	3.95	Included		[30]
Pseudomonas aeruginosa	DK1	2.11	Included		[31]
Pseudomonas aeruginosa	DK2	4.30	Excluded	Synonymous rate	[32]
Pseudomonas aeruginosa		154.50	Excluded	Hypermutator strains	[33]
Renibacterium salmoninarum		3.80	Included		[34]
Salmonella enterica	Kentucky	5.35	Included		[18]
Salmonella enterica	Typhi H58	1.78	Included		[18]
Salmonella enterica	paratyphi A	1.94	Included		[35]
Salmonella enterica	Agona	0.93	Included		[36]
Salmonella enterica	Typhimurium Lineage II	1.90	Included		[37]
Salmonella enterica	Typhimurium Lineage I	3.90	Included		[37]
Salmonella enterica	Typhimurium	3.35	Included		[38]
Salmonella enterica	Typhimurium	3.40	Included		[39]
Salmonella enterica	Enteritidis	100.00	Excluded	Hypermutator strain	[40]

Shigella dysenteriae	Sd1	8.70	Included		[41]
Shigella sonnei		6.00	Included		[42]
Staphylococcus aureus	ST93	4.50	Included		[43]
Staphylococcus aureus	ST239	16.00	Included		[44]
Staphylococcus aureus	CC398	16.80	Included		[45]
Staphylococcus aureus	ST22	13.00	Included		[46]
Staphylococcus aureus	USA300	12.20	Included		[47]
Staphylococcus aureus	USA300	12.50	Included		[48]
Staphylococcus aureus	ST239	32.50	Included		[49]
Staphylococcus aureus	ST239	33.00	Included		[50]
Staphylococcus aureus	ST239	37.90	Included		[51]
Staphylococcus aureus	ST225	20.00	Included		[52]
Staphylococcus aureus	MSSA	27.20	Included		[53]
Streptococcus agalactiae	CC1	6.40	Included		[54]
Streptococcus agalactiae	CC17	5.60	Included		[54]
Streptococcus agalactiae	CC19	9.30	Included		[54]
Streptococcus agalactiae	CC23	7.50	Included		[54]
Streptococcus equi		5.22	Included		[55]
Streptococcus pneumoniae	PMEN1	15.70	Included		[56]
Streptococcus pyogenes	Emm M1	8.06	Included		[57]
Streptococcus pyogenes	emm12	11.00	Included		[58]
Treponema pallidum		6.60	Included		[59]
Vibrio cholerae		9.60	Included		[18]
Vibrio cholerae		8.30	Included		[60]
Vibrio cholerae		2.35	Excluded	Synonymous rate	[61]
Yersinia pestis		0.07	Included		[62]
Yersinia pestis		0.20	Included		[63]
Yersinia pestis		0.16	Included		[18]
Yersinia pestis		0.23	Included		[18]
Yersinia pseudotuberculosis	ST19	3.87	Included		[64]

Yersinia pseudotuberculosis	ST43	5.63	Included	[64]
Yersinia pseudotuberculosis	ST9	20.10	Included	[64]
Yersinia pseudotuberculosis	ST42	3.57	Included	[64]
Yersinia pseudotuberculosis	ST14	8.67	Included	[64]

Supplementary Table 1. 81 estimates of the rate at which bacteria accumulate mutations per site per year (the accumulation rate) for 34 species of bacteria.

References

1. Holt K, Kenyon JJ, Hamidian M, Schultz MB, Pickard DJ, Dougan G, Hall RM. 2016 Five decades of genome evolution in the globally distributed, extensively antibiotic resistant Acinetobacter baumannii global clone 1. *Microb. Genomics* **2**. (doi:10.1099/mgen.0.000052)
2. Schultz MB *et al.* 2016 Repeated local emergence of carbapenem resistant Acinetobacter baumannii in a single hospital ward. *Microb. Genomics* **2**. (doi:10.1099/mgen.0.000050)
3. Bart MJ *et al.* 2014 Global Population Structure and Evolution of Bordetella pertussis and Their Relationship with Vaccination. *MBio* **5**, e01074-14. (doi:10.1128/mBio.01074-14.Editor)
4. Moran N a, McLaughlin HJ, Sorek R. 2009 The dynamics and time scale of ongoing genomic erosion in symbiotic bacteria. *Science* **323**, 379–382. (doi:10.1126/science.1167140)
5. Tamas I, Klasson L, Canbäck B, Näslund a K, Eriksson A-S, Wernegreen JJ, Sandström JP, Moran N a, Andersson SGE. 2002 50 Million Years of Genomic Stasis in Endosymbiotic Bacteria. *Science (80-.).* **296**, 2376–2379. (doi:10.1126/science.1071278)
6. Lieberman TD *et al.* 2011 Parallel bacterial evolution within multiple patients identifies candidate pathogenicity genes. *Nat. Genet.* **43**, 1275–1280. (doi:10.1038/ng.997)
7. Wilson DJ *et al.* 2009 Rapid evolution and the importance of recombination to the gastroenteric pathogen Campylobacter jejuni. *Mol. Biol. Evol.* **26**, 385–397. (doi:10.1093/molbev/msn264)
8. Read TD, Joseph SJ, Didelot X, Liang B, Patel L, Dean D. 2013 Comparative analysis of Chlamydia psittaci genomes reveals the recent emergence of a pathogenic lineage with a broad host range. *MBio* **4**, e00604-12. (doi:10.1128/mBio.00604-12)
9. Didelot X *et al.* 2012 Microevolutionary analysis of Clostridium difficile genomes to investigate transmission. *Genome Biol.* **13**, R118. (doi:10.1186/gb-2012-13-12-r118)
10. Steglich M, Nitsche A, von Müller L, Herrmann M, Kohl TA, Niemann S, Nübel U. 2015 Tracing the Spread of Clostridium difficile Ribotype 027 in Germany Based on Bacterial Genome Sequences. *PLoS One* **10**, e0139811. (doi:10.1371/journal.pone.0139811)
11. He M *et al.* 2013 Emergence and global spread of epidemic healthcare-associated Clostridium difficile. *Nat. Genet.* **45**, 109–13. (doi:10.1038/ng.2478)
12. Howden B, Holt K, Lam M, Seemann T. 2013 Genomic Insights Howden, B., Holt, K., Lam, M., & Seemann, T. (2013). Genomic Insights to Control the Emergence of Vancomycin-Resistant

Enterococci. mBio. doi:10.1128/mBio.00412-13.Editorto Control the Emergence of Vancomycin-Resistant Enterococci. *MBio* **4**, e00412-13. (doi:10.1128/mBio.00412-13.Editor)

13. Reeves PR *et al.* 2011 Rates of Mutation and Host Transmission for an Escherichia coli Clone over 3 Years. *PLoS One* **6**, e26907. (doi:10.1371/journal.pone.0026907)
14. Falush D, Kraft C, Taylor NS, Correa P, Fox JG, Achtman M, Suerbaum S. 2001 Recombination and mutation during long-term gastric colonization by Helicobacter pylori : Estimates of clock rates , recombination size , and minimal age. *Proc. Natl. Acad. Sci. U. S. A.* **98**, 15056–15061.
15. Morelli G, Didelot X, Kusecek B, Schwarz S, Bahlawane C, Falush D, Suerbaum S, Achtman M. 2010 Microevolution of Helicobacter pylori during prolonged infection of single hosts and within families. *PLoS Genet.* **6**, e1001036. (doi:10.1371/journal.pgen.1001036)
16. Didelot X, Nell S, Yang I, Woltemate S, van der Merwe S, Suerbaum S. 2013 Genomic evolution and transmission of Helicobacter pylori in two South African families. *Proc. Natl. Acad. Sci. U. S. A.* **110**, 13880–5. (doi:10.1073/pnas.1304681110)
17. Kennemann L *et al.* 2011 Helicobacter pylori genome evolution during human infection. *Proc. Natl. Acad. Sci. U. S. A.* **108**, 5033–5038. (doi:10.1073/pnas.1018444108)
18. Duchêne S, Holt KE, Weill F-X, Le Hello S, Hawkey J, Edwards DJ, Fourment M, Holmes EC. 2016 Genome-scale rates of evolutionary change in bacteria. *Microb. Genomics* **2**. (doi:10.1101/069492)
19. Sánchez-Busó L, Comas I, Jorques G, González-Candelas F. 2014 Recombination drives genome evolution in outbreak-related Legionella pneumophila isolates. *Nat. Genet.* **46**, 1205–1211. (doi:10.1038/ng.3114)
20. Bryant JM *et al.* 2013 Whole-genome sequencing to identify transmission of Mycobacterium abscessus between patients with cystic fibrosis: a retrospective cohort study. *Lancet* **381**, 1551–1560. (doi:10.1016/S0140-6736(13)60632-7)
21. Biek R *et al.* 2012 Whole Genome Sequencing Reveals Local Transmission Patterns of Mycobacterium bovis in Sympatric Cattle and Badger Populations. *PLoS Pathog.* **8**, e1003008. (doi:10.1371/journal.ppat.1003008)
22. Schuenemann VJ *et al.* 2013 Genome-Wide Comparison of Medieval and Modern Mycobacterium leprae. *Science (80-.).* **341**, 179–183. (doi:10.1126/science.1238286)
23. Bos KI *et al.* 2014 Pre-Columbian mycobacterial genomes reveal seals as a source of New World human tuberculosis. *Nature* **514**, 494–497. (doi:10.1038/nature13591)
24. Ford CB *et al.* 2011 Use of whole genome sequencing to estimate the mutation rate of Mycobacterium tuberculosis during latent infection. *Nat. Genet.* **43**, 482–486. (doi:10.1038/ng.811)
25. Walker TM *et al.* 2013 Whole-genome sequencing to delineate Mycobacterium tuberculosis outbreaks: a retrospective observational study. *Lancet Infect. Dis.* **13**, 137–146. (doi:10.1016/S1473-3099(12)70277-3)
26. Roetzer A *et al.* 2013 Whole Genome Sequencing versus Traditional Genotyping for Investigation of a Mycobacterium tuberculosis Outbreak: A Longitudinal Molecular Epidemiological Study. *PLoS Med.* **10**, e1001387. (doi:10.1371/journal.pmed.1001387)
27. Vandelannoote K *et al.* 2017 Multiple introductions and recent spread of the emerging human pathogen Mycobacterium ulcerans across Africa. *Genome Biol. Evol.* **9**, 414–426. (doi:10.1093/gbe/evx003)
28. Delaney NF, Balenger S, Bonneaud C, Marx CJ, Hill GE, Ferguson-Noel N, Tsai P, Rodrigo A, Edwards S V. 2012 Ultrafast Evolution and Loss of CRISPRs Following a Host Shift in a Novel

Wildlife Pathogen, Mycoplasma gallisepticum. *PLoS Genet.* **8**, e1002511. (doi:10.1371/journal.pgen.1002511)
29. Grad YH *et al.* 2014 Genomic epidemiology of Neisseria gonorrhoeae with reduced susceptibility to cefixime in the USA: a retrospective observational study. *Lancet Infect. Dis.* **14**, 220–6. (doi:10.1016/S1473-3099(13)70693-5)
30. Marvig RL, Johansen HK, Molin S, Jelsbak L. 2013 Genome analysis of a transmissible lineage of pseudomonas aeruginosa reveals pathoadaptive mutations and distinct evolutionary paths of hypermutators. *PLoS Genet.* **9**, e1003741. (doi:10.1371/journal.pgen.1003741)
31. Markussen T, Marvig RL. 2014 Environmental Heterogeneity Drives Within-Host Diversification and Evolution of Pseudomonas aeruginosa. *MBio* **5**, e01592-14. (doi:10.1128/mBio.01592-14.Editor)
32. Yang L *et al.* 2011 Evolutionary dynamics of bacteria in a human host environment. *Proc. Natl. Acad. Sci.* **108**, 7481–7486. (doi:10.1073/pnas.1018249108)
33. Feliziani S, Marvig RL, Luján AM, Moyano AJ, Di Rienzo JA, Krogh Johansen H, Molin S, Smania AM. 2014 Coexistence and Within-Host Evolution of Diversified Lineages of Hypermutable Pseudomonas aeruginosa in Long-term Cystic Fibrosis Infections. *PLoS Genet.* **10**, e1004651. (doi:10.1371/journal.pgen.1004651)
34. Brynildsrud O *et al.* 2014 Microevolution of Renibacterium salmoninarum: evidence for intercontinental dissemination associated with fish movements. *ISME J.* **8**, 746–56. (doi:10.1038/ismej.2013.186)
35. Zhou Z, McCann a., Weill F-X, Blin C, Nair S, Wain J, Dougan G, Achtman M. 2014 Transient Darwinian selection in Salmonella enterica serovar Paratyphi A during 450 years of global spread of enteric fever. *Proc. Natl. Acad. Sci.* **111**, 12199–12204. (doi:10.1073/pnas.1411012111)
36. Zhou Z *et al.* 2013 Neutral Genomic Microevolution of a Recently Emerged Pathogen, Salmonella enterica Serovar Agona. *PLoS Genet.* **9**, e1003471. (doi:10.1371/journal.pgen.1003471)
37. Okoro CK *et al.* 2012 Intracontinental spread of human invasive Salmonella Typhimurium pathovariants in sub-Saharan Africa. *Nat. Genet.* **44**, 1215–1221. (doi:10.1038/ng.2423)
38. Hawkey J, Edwards DJ, Dimovski K, Hiley L, Billman-Jacobe H, Hogg G, Holt KE. 2013 Evidence of microevolution of Salmonella Typhimurium during a series of egg-associated outbreaks linked to a single chicken farm. *BMC Genomics* **14**, 800. (doi:10.1186/1471-2164-14-800)
39. Mather AE. 2013 Distinguishable Epidemics of Multidrug-Resistant Salmonella Typhimurium DT104 in Different Hosts. *Science (80-.).* **341**, 1514–1518.
40. Klemm EJ *et al.* 2016 Emergence of host-adapted Salmonella Enteritidis through rapid evolution in an immunocompromised host. *Nat. Microbiol.* **1**, 15023. (doi:10.1038/nmicrobiol.2015.23)
41. Njamkepo E *et al.* 2016 Global phylogeography and evolutionary history of Shigella dysenteriae type 1. *Nat. Microbiol.* , 16027. (doi:10.1038/nmicrobiol.2016.27)
42. Holt KE *et al.* 2012 Shigella sonnei genome sequencing and phylogenetic analysis indicate recent global dissemination from Europe. *Nat. Genet.* **44**, 1056–1059. (doi:10.1038/ng.2369)
43. Stinear TP, Holt KE, Chua K, Stepnell J, Tuck KL, Coombs G, Harrison PF, Seemann T, Howden BP. 2014 Adaptive change inferred from genomic population analysis of the ST93 epidemic clone of community-associated methicillin-resistant Staphylococcus aureus. *Genome Biol. Evol.* **6**, 366–378. (doi:10.1093/gbe/evu022)

44. Baines SL *et al.* 2015 Convergent adaptation in the dominant global hospital clone ST239 of methicillin-resistant Staphylococcus aureus. *MBio* **6**, e00080-15. (doi:10.1128/mBio.00080-15)
45. Ward MJ, Gibbons CL, McAdam PR, van Bunnik BAD, Girvan EK, Edwards GF, Fitzgerald JR, Woolhouse MEJ. 2014 Time-scaled evolutionary analysis of the transmission and antibiotic resistance dynamics of Staphylococcus aureus clonal complex 398. *Appl. Environ. Microbiol.* **80**, 7275–7282. (doi:10.1128/AEM.01777-14)
46. Holden MTG *et al.* 2013 A genomic portrait of the emerfences, evolution, and global spread of methicillin-resistant Staphylococcus aureus. *Genome Res.* **23**, 653–664. (doi:10.1101/gr.147710.112.Freely)
47. Uhlemann A-C, Dordel J, Knox JR, Raven KE, Parkhill J, Holden MTG, Peacock SJ, Lowy FD. 2014 Molecular tracing of the emergence, diversification, and transmission of S. aureus sequence type 8 in a New York community. *Proc. Natl. Acad. Sci.* **111**, 6738–6743. (doi:10.1073/pnas.1401006111)
48. Alam MT, Read TD, Petit RA, Boyle-Vavra S, Miller LG, Eells SJ, Daum RS, David MZ. 2015 Transmission and Microevolution of USA300 MRSA in U.S. Households: Evidence from Whole-Genome Sequencing. *MBio* **6**, e00054-15. (doi:10.1128/mBio.00054-15)
49. Smyth DS, McDougal LK, Gran FW, Manoharan A, Enright MC, Song JH, De Lencastre H, Robinson DA. 2010 Population structure of a hybrid clonal group of methicillin-resistant Staphylococcus aureus, ST239-MRSA-III. *PLoS One* **5**, e8582. (doi:10.1371/journal.pone.0008582)
50. Harris SR *et al.* 2010 Evolution of MRSA During Hospital Transmission and Intercontinental Spread. *Science (80-.).* **327**, 469–474. (doi:10.1126/science.1182395)
51. Gray RR, Tatem AJ, Johnson J a., Alekseyenko A V., Pybus OG, Suchard M a., Salemi M. 2011 Testing spatiotemporal hypothesis of bacterial evolution using methicillin-resistant staphylococcus aureus ST239 genome-wide data within a bayesian framework. *Mol. Biol. Evol.* **28**, 1593–1603. (doi:10.1093/molbev/msq319)
52. Nübel U *et al.* 2010 A Timescale for Evolution, Population Expansion, and Spatial Spread of an Emerging Clone of Methicillin-Resistant Staphylococcus aureus. *PLoS Pathog.* **6**, e1000855. (doi:10.1371/journal.ppat.1000855)
53. Young BC *et al.* 2012 Evolutionary dynamics of Staphylococcus aureus during progression from carriage to disease. *Proc. Natl. Acad. Sci. U. S. A.* **109**, 4550–4555. (doi:10.1073/pnas.1113219109)
54. Da Cunha V *et al.* 2014 Streptococcus agalactiae clones infecting humans were selected and fixed through the extensive use of tetracycline. *Nat. Commun.* **5**, 4544. (doi:10.1038/ncomms5544)
55. Harris SR, Robinson C, Steward KF, Webb KS, Paillot R, Parkhill J, Holden MTG, Waller AS. 2015 Genome specialization and decay of the strangles pathogen, Streptococcus equi, is driven by persistent infection. *Genome Res.* **25**, 1360–1371. (doi:10.1101/014118)
56. Croucher NJ *et al.* 2011 Rapid pneumococcal evolution in response to clinical interventions. *Science (80-.).* **331**, 430–4. (doi:10.1126/science.1198545)
57. Nasser W *et al.* 2014 Evolutionary pathway to increased virulence and epidemic group A Streptococcus disease derived from 3,615 genome sequences. *Proc. Natl. Acad. Sci.* **111**, E1768–E1776. (doi:10.1073/pnas.1403138111)
58. Davies MR *et al.* 2015 Emergence of scarlet fever Streptococcus pyogenes emm12 clones in

Hong Kong is associated with toxin acquisition and multidrug resistance. *Nat. Genet.* **47**, 84–7. (doi:10.1038/ng.3147)

59. Arora N *et al.* 2016 Origin of modern syphilis and emergence of a contemporary pandemic cluster. *Nat. Micro* **2**, e051037. (doi:10.1101/051037)
60. Mutreja A *et al.* 2011 Evidence for several waves of global transmission in the seventh cholera pandemic. *Nature* **477**, 462–465. (doi:10.1038/nature10392)
61. Feng L *et al.* 2008 A recalibrated molecular clock and independent origins for the cholera pandemic clones. *PLoS One* **3**, e4053. (doi:10.1371/journal.pone.0004053)
62. Morelli G *et al.* 2010 Yersinia pestis genome sequencing identifies patterns of global phylogenetic diversity. *Nat. Genet.* **42**, 1140–1143. (doi:10.1038/ng.705)
63. Bos KI *et al.* 2011 A draft genome of Yersinia pestis from victims of the Black Death. *Nature* **478**, 506–510. (doi:10.1038/nature10549)
64. Williamson DA *et al.* 2017 Genomic insights into a sustained national outbreak of Yersinia pseudotuberculosis. *Genome Biol. Evol.* **8**, 3806–3814. (doi:10.1093/gbe/evw285)

Species	Mutation Rate/ site/generation (x10^{-10})	Reference
Agrobacterium tumefaciens	2.92	[1]
Arthrobacter sp	3.18	[2]
Bacillus subtilis	3.28	[3]
Burkholderia cenocepacia	1.33	[4]
Caulobacter crescentus	3.46	[2]
Colwellia psychrerythraea	8.38	[2]
Deinococcus radiodurans	4.99	[5]
Escherichia coli	2.54	[6,7]
Flavobacterium sp	3.91	[2]
Gemmata obscuriglobus	2.38	[2]
Janthinobacterium lividum	1.22	[2]
Kineococcus radiotolerans	3.9	[2]
Lactococcus lactis	16.6	[2]
Mesoplasma florum	97.8	[8]
Micrococcus sp	3.18	[2]
Mycobacterium smegmatis	5.27	[9]
Pseudomonas aeruginosa	0.792	[10]
Rhodobacter sphaeroides	1.17	[2]
Ruegeria pomeroyi	1.39	[11]
Salmonella enterica	7	[12]
Staphylococcus aureus	4.38	[2]
Staphylococcus epidermidis	7.4	[1]
Teredinibacter turnerae	11.4	[13]
Vibrio cholerae	1.07	[14]
Vibrio fischeri	2.07	[14]
Vibrio shilonii	2.29	[15]

Supplementary Table 2. Estimates of the mutation rate per site per generation for 26 species of bacteria.

References

1. Sung W, Ackerman MS, Dillon MM, Platt TG, Fuqua C, Cooper VS, Lynch M. 2016 Evolution of the Insertion-Deletion Mutation Rate Across the Tree of Life. *G3* **6**, 2583–2591. (doi:10.1534/g3.116.030890)
2. Long H *et al.* 2018 Evolutionary determinants of genome-wide nucleotide composition. *Nat. Ecol. Evol.* **2**, 1–4. (doi:10.1038/s41559-017-0425-y)
3. Sung W, Ackerman MS, Miller SF, Williams E, Foster PL, Lynch M. 2015 Asymmetric Context-Dependent Mutation Patterns Revealed through Mutation – Accumulation Experiments Article Fast Track. *Mol. Biol. Evol.* **32**, 1672–1683. (doi:10.1093/molbev/msv055)
4. Dillon MM, Sung W, Lynch M, Cooper VS, Cooper V. 2015 The rate and molecular spectrum of spontaneous mutations in the GC-rich multi- chromosome genome of Burkholderia cenocepacia. *Genetics* **200**, 935–946. (doi:10.1534/genetics.115.176834)
5. Long H, Kucukyildirim S, Sung W, Williams E, Lee H, Ackerman M, Doak TG, Tang H, Lynch M. 2015 Background mutational features of the radiation-resistant bacterium Deinococcus radiodurans. *Mol. Biol. Evol.* **32**, 2383–2392. (doi:10.1093/molbev/msv119)
6. Lee H, Popodi E, Tang H, Foster PL. 2012 PNAS Plus: Rate and molecular spectrum of spontaneous mutations in the bacterium Escherichia coli as determined by whole-genome

sequencing. *Proc. Natl. Acad. Sci.* **109**, E2774–E2783. (doi:10.1073/pnas.1210309109)
7. Foster PL, Lee H, Popodi E, Townes JP, Tang H. 2015 Determinants of spontaneous mutation in the bacterium Escherichia coli as revealed by whole-genome sequencing. *Proc. Natl. Acad. Sci.* **112**, E5990–E5999. (doi:10.1073/pnas.1512136112)
8. Sung W, Ackerman MS, Miller SF, Doak TG, Lynch M. 2012 Drift-barrier hypothesis and mutation-rate evolution. *Proc. Natl. Acad. Sci. U. S. A.* **109**, 18488–92. (doi:10.1073/pnas.1216223109)
9. Kucukyildirim S, Long H, Sung W, Miller SF, Doak TG, Lynch M. 2016 The Rate and Spectrum of Spontaneous Mutations in Mycobacterium smegmatis , a Bacterium Naturally Devoid of the Postreplicative Mismatch Repair Pathway. *G3* **6**, 2157–2163. (doi:10.1534/g3.116.030130)
10. Dettman JR, Sztepanacz JL, Kassen R. 2016 The properties of spontaneous mutations in the opportunistic pathogen Pseudomonas aeruginosa. *BMC Genomics* , 1–14. (doi:10.1186/s12864-015-2244-3)
11. Sun Y, Powell KE, Sung W, Lynch M, Moran MA, Luo H. 2017 Spontaneous mutations of a model heterotrophic marine bacterium. *ISME J.* **11**, 1713–1718. (doi:10.1038/ismej.2017.20)
12. Lind P a, Andersson DI. 2008 Whole-genome mutational biases in bacteria. *Proc. Natl. Acad. Sci. U. S. A.* **105**, 17878–17883. (doi:10.1073/pnas.0804445105)
13. Senra MVX, Sung W, Ackerman M, Miller SF, Lynch M, Soares CAG. 2018 An unbiased genome-wide view of the mutation rate and spectrum of the endosymbiotic bacterium Teredinibacter turnerae. *Genome Biol. Evol.* **10**, 723–730. (doi:10.1093/gbe/evy027)
14. Dillon MM, Sung W, Sebra R, Lynch M, Cooper V. 2016 Genome-wide biases in the rate and molecular spectrum of spontaneous mutations in Vibrio cholerae and Vibrio fischeri. *Mol. Biol. Evol.* **34**, 93–109. (doi:10.1093/molbev/msw224)
15. Strauss C, Long H, Patterson CE, Te R, Lynch M. 2017 Genome-wide mutation rate response to pH change in the coral reef. *MBio* **8**, e01021-17.

Species	dN/dS	Reference
Buchnera aphidicola	0.125	[1]
Burkholderia dolosa	1	[2]
Helicobacter pylori	0.14	[3]
Mycoplasma gallisepticum	0.2	[4]
Pseudomonas aeruginosa DK1	0.56	[5]
Pseudomonas aeruginosa DK2	0.66	[6]
Pseudomonas aeruginosa DK2	0.79	[7]
Salmonella enterica Agona	0.67	[8]
Salmonella enterica Paratyphi A	0.8	[9]
Salmonella enterica Typhimurium	0.52	[10]
Staphylococcus aureus (ST225)	0.77	[11]
Streptococcus equi	0.6	[12]

Supplementary Table 3. dN/dS values for 8 species of bacteria

References

1. Moran N a, McLaughlin HJ, Sorek R. 2009 The dynamics and time scale of ongoing genomic erosion in symbiotic bacteria. *Science* **323**, 379–382. (doi:10.1126/science.1167140)
2. Lieberman TD *et al.* 2011 Parallel bacterial evolution within multiple patients identifies candidate pathogenicity genes. *Nat. Genet.* **43**, 1275–1280. (doi:10.1038/ng.997)
3. Didelot X, Nell S, Yang I, Woltemate S, van der Merwe S, Suerbaum S. 2013 Genomic evolution and transmission of Helicobacter pylori in two South African families. *Proc. Natl. Acad. Sci. U. S. A.* **110**, 13880–5. (doi:10.1073/pnas.1304681110)
4. Delaney NF, Balenger S, Bonneaud C, Marx CJ, Hill GE, Ferguson-Noel N, Tsai P, Rodrigo A, Edwards S V. 2012 Ultrafast Evolution and Loss of CRISPRs Following a Host Shift in a Novel Wildlife Pathogen, Mycoplasma gallisepticum. *PLoS Genet.* **8**, e1002511. (doi:10.1371/journal.pgen.1002511)
5. Markussen T, Marvig RL. 2014 Environmental Heterogeneity Drives Within-Host Diversification and Evolution of Pseudomonas aeruginosa. *MBio* **5**, e01592-14. (doi:10.1128/mBio.01592-14.Editor)
6. Marvig RL, Johansen HK, Molin S, Jelsbak L. 2013 Genome analysis of a transmissible lineage of pseudomonas aeruginosa reveals pathoadaptive mutations and distinct evolutionary paths of hypermutators. *PLoS Genet.* **9**, e1003741. (doi:10.1371/journal.pgen.1003741)
7. Yang L *et al.* 2011 Evolutionary dynamics of bacteria in a human host environment. *Proc. Natl. Acad. Sci.* **108**, 7481–7486. (doi:10.1073/pnas.1018249108)
8. Zhou Z *et al.* 2013 Neutral Genomic Microevolution of a Recently Emerged Pathogen, Salmonella enterica Serovar Agona. *PLoS Genet.* **9**, e1003471. (doi:10.1371/journal.pgen.1003471)
9. Zhou Z, McCann a., Weill F-X, Blin C, Nair S, Wain J, Dougan G, Achtman M. 2014 Transient Darwinian selection in Salmonella enterica serovar Paratyphi A during 450 years of global spread of enteric fever. *Proc. Natl. Acad. Sci.* **111**, 12199–12204.

(doi:10.1073/pnas.1411012111)
10. Hawkey J, Edwards DJ, Dimovski K, Hiley L, Billman-Jacobe H, Hogg G, Holt KE. 2013 Evidence of microevolution of Salmonella Typhimurium during a series of egg-associated outbreaks linked to a single chicken farm. *BMC Genomics* **14**, 800. (doi:10.1186/1471-2164-14-800)
11. Nübel U *et al.* 2010 A Timescale for Evolution, Population Expansion, and Spatial Spread of an Emerging Clone of Methicillin-Resistant Staphylococcus aureus. *PLoS Pathog.* **6**, e1000855. (doi:10.1371/journal.ppat.1000855)
12. Harris SR, Robinson C, Steward KF, Webb KS, Paillot R, Parkhill J, Holden MTG, Waller AS. 2015 Genome specialization and decay of the strangles pathogen, Streptococcus equi, is driven by persistent infection. *Genome Res.* **25**, 1360–1371. (doi:10.1101/014118)

δs	δi	δn	mu_obs/mu_true
1	0.9	0.8	0.87
""	""	0.6	0.75
""	""	0.3	0.57
""	0.8	0.8	0.85
""	""	0.6	0.73
""	""	0.3	0.55
""	0.4	0.8	0.79
""	""	0.6	0.67
""	""	0.3	0.49
0.5	0.45	0.4	0.43
""	""	0.3	0.37
""	""	0.15	0.28
""	0.4	0.4	0.43
""	""	0.3	0.37
""	""	0.15	0.28
""	0.2	0.4	0.40
""	""	0.3	0.34
""	""	0.15	0.25

Supplementary Table 4. Testing different parameter combinations to investigate how sensitive the doubling time estimate is to the parameters in equation 1 in the main text. Each parameter is varied in turn. δi and δn are dependent on δs so they are halved when δs is halved.
