# Supplementary material for: The distribution of bacterial doubling times in the wild
Source: Proc Biol Sci. 2018 Jun 13;285(1880):20180789. doi: 10.1098/rspb.2018.0789 (PMC6015860; doi:10.1098/rspb.2018.0789)
Supplement: Electronic supplementary material (ESM) titles and captions [file rspb20180789supp2.docx]

**Electronic supplementary material (ESM) titles and captions**

**Supplementary Table 1. Accumulation Rates**

81 estimates of the rate at which bacteria accumulate mutations per site per year (the accumulation rate) for 34 species of bacteria.

**Supplementary Table 2. Mutation Rates**

Estimates of the mutation rate per site per generation for 26 species of bacteria.

**Supplementary Table 3.** **dN/dS values**

dN/dS values for 8 species of bacteria

**Supplementary Table 4.**

Testing different parameter combinations to investigate how sensitive the doubling time estimate is to the parameters in equation 1 in the main text.

**Supplementary Figure 1. Mutation rate vs genome size**

The mutation rate vs genome size plotted for 26 species of bacteria.

**Supplementary Figure 2.** 16s rRNA phylogenies

16s rRNA phylogenies for species for which we have a mutation rate estimate (A,B) and an accumulation rate (C). When all 26 species are included for the mutation rate data (A) Flavobacterium sp and the Alphaproteobacteria are erroneously positioned with the gram positive bacteria. This is resolved after exclusion of Flavobacterium sp and Gemmata obscuriglobus. (B).
